# Global denoising for 3D MRI

**DOI:** 10.1186/s12938-016-0168-z

**Published:** 2016-05-12

**Authors:** Xi Wu, Zhipeng Yang, Jing Peng, Jiliu Zhou

**Affiliations:** Department of Computer Science, Chengdu University of Information Technology, No.24 Block 1, Xuefu Road, Chengdu, 610225 People’s Republic of China

**Keywords:** Global denoising, Nyström method, 3D MRI, k-means clustering

## Abstract

**Background:**

Denoising is the primary preprocessing step for subsequent application of MRI. However, most commonly-used patch-based denoising methods are heavily dependent on the degree of patch matching. Due to the large number of voxels in the 3D MRI dataset, the procedure of searching sufficient similarity patches was limited by the empirical compromising between computational efficiency and estimation accuracy, and cannot fulfill the application in multimodal MRI dataset with different SNR and resolutions.

**Methods:**

In this study, we propose a modified global filtering framework for 3D MRI. For each denoising voxel, the similarity weighting matrix is computed using the reference patch and other patches from the whole dataset. This large weighting matrix is then approximated using the k-means clustering Nyström method to achieve computational viability.

**Results:**

Experiments on both synthetic and in vivo MRI datasets demonstrated that the proposed adaptive Nyström low-rank approximation could achieve competitive estimation compared with exact global filter while reducing the sampling rate by four orders of magnitude. In addition, the corresponding global filter improved patches-based method in both spatial and transform domain.

**Conclusion:**

We propose a global denoising framework for 3D MRI which extracts information from the entire dataset to restore each voxel. This large weighting matrix of the global filter is approximated using Nyström low-rank approximation with an adaptive k-means clustering sampling scheme, which significantly reduce the sampling rate as well as the running time. The proposed method is capable of denoising in multimodal MRI dataset and can be used to improve currently used patch-based methods.

## Background

Magnetic resonance imaging is of ever increasing importance in clinical and research settings, owing to its accurate non-invasive 3D representation of the internal human structure. Nevertheless, MRI suffers significantly from noise arising during the acquisition procedure. Hence, denoising is used as the primary preprocessing step for subsequent clinical diagnosis and other applications, such as registration and segmentation.

A wide range of denoising methods have been proposed to estimate the latent image from noisy observations and many of them can be categorized as patch-based filters. In general, patch-based filters estimate each voxel in an MRI dataset using patches selected according to some predefined similarity criteria. For example, a non-local means filter [1] denoises images by minimizing the penalty term for the weighted distance between filtered image patches and other patches in the search window. This weighted distance was first defined as a decreasing function of the Euclidean distance between gray level intensities in the patches, and then adapted to various high-order operators to enhance the performance [2–5]. This strategy has also been implemented effectively in statistical methods, such as expectation–maximization-based Bayesian estimation [6] and maximum likelihood estimation [7] to promote more effective denoising of 3D MRI. On the other hand, based on the assumption that latent images have a sparse representation in some transform domain, numerous transforms have been proposed to filter noisy images. These include the discrete cosine transform (DCT) [8], principal component analysis [9], over-complete dictionaries [10], and high-order singular value decomposition [11]. The filtering process was implemented by applying a shrinkage function to the transform coefficients and recovering estimated patches with an inverse transform. For instance, the block matching in three dimensions (BM3D) method [8] collects a group of similar patches from the reference patches to construct a 3D array. After projecting the 3D array onto a 3D transform basis, the coefficients are truncated by a hard threshold and filtered patches are reconstructed by inversion of the transform.

Although promising results have been achieved, the performance of patch-based filters is still limited by the necessity of finding sufficiently similar patches. Specifically, if the similar patches cannot be effectively represented, the denoising process will be affected by mistaken truncating of the signal component or some other implementation due to a lack of redundancy provided by similar patches. Since the number of voxels of 3D MRI dataset is large, most of the currently used patch-based methods implemented a subset of the whole dataset as the searching window and compromise between computational efficiency and estimation accuracy empirically [2–4]. However, these predefined parameters cannot be used to fulfill various MRI dataset, especially when multimodal MRI has different signal to noise ratio (SNR) or resolutions.

Recently, another group of methods stems from the emerging theory of low-rank matrix completion, which is derived from compressed sensing theory [12], were proposed to accomplish the accurate restoration from the whole 3D dataset effectively. Low-rank matrix completion technology relies on self-similarities across different slices or frames in an MRI dataset to construct a low-rank matrix and demonstrates significant benefits for various MRI applications, including reconstruction from highly under-sampled k-t space data [13], super-resolution [14], and denoising [15–17]. Lin exploited the self-similarity between multi-channel coil images and formulated denoising as a non-smooth convex optimization problem [15]. Lam incorporated low-rank modeling and an edge-preserving smoothing algorithm prior to denoising MRI data in a unified mathematical framework [16]. Recently, Talebi and Milafar [18] proposed a promising global approach based on low-rank approximation to denoise natural scene images. Rather than empirically predefining a part of the image as a search window, this global filter introduced the whole dataset as a search window and implemented a low-rank Nyström extension to approximate the large weighting matrix for practical applications [19].

In this study, we propose a modified global filtering framework for 3D MRI. The proposed framework estimates denoised voxels based on a similarity weighting matrix which is computed between the reference patches and other patches located throughout the entire dataset. Meanwhile, the large similarity weighting matrix is low-rank approximated using the Nyström method [19] to achieve computational viability. The contributions of this work are as follows: firstly, the global filtering framework is extended and applied to 3D MRI datasets. More importantly, since the computational burden is prohibitively high due to the sophisticated structure of 3D MRI data and other MRI modalities such as diffusion weighted imaging (DWI) [20], a simple and efficient sampling scheme, k-means clustering, is introduced to improve the Nyström method for applicable implementations. This adaptive sampling scheme enhances the matrix estimation accuracy and effectively improves the computational efficiency. This is significantly beneficial for global filtering.

In the remainder of this paper, we describe our global filtering framework in detail, together with the adaptive Nyström low-rank approximation. After quantitative and qualitative comparisons of the proposed global framework for multimodal MRI datasets, we consider the advantages and limitations of our proposed technique.

## Methods

### General model of iterative global denoising

Image denoising, as the most common image restoration problem, can be modeled as:

1 $${\mathbf{y}} = {\mathbf{x}} + {\mathbf{n}},$$

where **y** and **x** are column vectors in the noisy image [*y*_1_, *y*_2_, …, *y*_*n*_]^T^ and the ‘original’ image [*x*_1_, *x*_2_, …, *x*_*n*_]^T^ and **n** represents additive Gaussian noise. The commonly-used spatial domain denoising method can be represented using the following restoration framework:

2 $${\hat{\mathbf{x}}}_{i} = \arg \mathop {\hbox{min} }\limits_{{{\mathbf{x}}_{i} }} \sum\limits_{j = 1}^{n} {[{\mathbf{x}}_{i} - {\mathbf{y}}_{j} ]^{2} K_{ij} } ,$$

where the kernel *K*_*ij*_ weights the similarity between **x**_*i*_ and **y**_*j*_ and ${\hat{\mathbf{x}}}_{i}$ is the estimated voxel.

This equation can be minimized by substituting the normalized weighting sum of each voxel:

3 $${\hat{\mathbf{x}}}_{i} = {\mathbf{w}}_{i}^{T} {\mathbf{y}} ,$$

where the weighting vector can be defined as

4 $${\mathbf{w}}_{i} = \frac{1}{{\sum\nolimits_{j = 1}^{n} {M_{ij} } }}[M_{i1} ,M_{i2} , \ldots ,M_{in} ]^{T} ,$$

in which $[M_{i1} ,M_{i2} , \ldots ,M_{in} ]$ denotes the *i*th row of the kernel **M**. The global denoising process for the whole image can be represented by stacking the weighting vectors:

5 $${\hat{\mathbf{x}}} = \left[ \begin{array}{c} {\mathbf{w}}_{1}^{T} \hfill \\ {\mathbf{w}}_{2}^{T} \hfill \\ \vdots \hfill \\ {\mathbf{w}}_{i}^{T} \hfill \\ \end{array} \right]{\mathbf{y}} = {\mathbf{Wy}},$$

where **W** is the denoising weighting matrix used to estimate the denoised voxel ${\hat{\mathbf{x}}}$.

While **W** is not generally a symmetric matrix, it can be closely approximated as a symmetric, positive definite matrix [21] and then eigen-decomposed as follows:

6 $${\mathbf{W}} = {\mathbf{VEV}}^{T} ,$$

where ${\mathbf{V}} = [{\mathbf{v}}_{1} , \ldots ,{\mathbf{v}}_{n} ]$ is a complete orthonormal basis for ${\mathbb{R}}^{n}$. ${\mathbf{E}} = diag[\lambda_{1} , \ldots ,\lambda_{n} ]$ contains the eigenvalues in decreasing order $0 \le \lambda_{n} \le \cdots \le \lambda_{1} = 1$. The global denoising proposed in (5) can then be rewritten as:

7 $${\hat{\mathbf{x}}} = {\mathbf{Wy}} = {\mathbf{VEV}}^{T} {\mathbf{y}} ,$$

In the proposed global denoising framework, **y** is first projected onto the eigenvector of **W**; then the projected signal is manipulated with its corresponding eigenvalue, which maps back to the original domain to obtain the final estimation ${\hat{\mathbf{x}}}$. Since the filter **W** was performed on the leading eigenvector, the mean square error (MSE) of the global filter truncated with *m* leading eigenvalues can be computed as [22]:

8 $$MSE(m) = \sum\limits_{i = 1}^{n} {x_{i}^{2} + \sum\limits_{j = 1}^{m} {((\lambda_{j}^{2} - 2\lambda_{j} )b_{j}^{2} + \sigma^{2} \lambda_{j}^{2} )} } ,$$

where ${\mathbf{b}} = {\mathbf{V}}^{T} {\mathbf{x}} = [b_{1} , \ldots ,b_{n} ]^{T}$ contains the projected signal in all modes. However, as indicated in [16], hard threshold truncating preserves an accurate estimation of the optimal threshold, which can be used to avoid under- or over-smoothing. In this study, we introduce an iterative diffusion model [22] which can tune the filter to vary its filtering strength iteratively. The proposed global filter can then be rewritten as:

9 $${\hat{\mathbf{x}}} = \widehat{{\mathbf{W}}}{\mathbf{y}} = {\mathbf{V}}_{m} {\mathbf{E}}_{m}^{k} {\mathbf{V}}_{m}^{T} {\mathbf{y}} ,$$

where ${\mathbf{V}}_{m} = [{\mathbf{v}}_{1} ,{\mathbf{v}}_{2} , \ldots {\mathbf{v}}_{m} ]$, ${\mathbf{S}}_{m}^{k} = diag[\lambda_{1}^{k} ,\lambda_{2}^{k} , \ldots ,\lambda_{m}^{k} ]$, and *k* denotes the iteration number. When this model is used, the mean square error (MSE) can be rewritten for this iterative truncated filter as:

10 $$MSE(k,m) = \sum\limits_{i = 1}^{n} {x_{i}^{2} + \sum\limits_{j = 1}^{m} {((\lambda_{j}^{2k} - 2\lambda_{j}^{k} )b_{j}^{2} + \sigma^{2} \lambda_{j}^{2k} )} } ,$$

The minimization problem can then be extended to estimate the shrinkage ($\widehat{k}$) and truncation ($\widehat{m}$) factors from an estimation of the mean square error.

It is worth noting that the initial global filter proposed in (9) was designated for images corrupted by additive Gaussian noise and cannot be directly applied to MRI datasets which are corrupted by Rician noise [23]. Owing to advantages of the recently-proposed method of variance-stabilizing transforms for the Rician distribution [22], the global filter in (9) can be successfully applied to Rician noise data without any modification of the algorithm [22, 24]. The VST transform can effectively remove the dependence of noise variance for the underlying signal before denoising and can compensate for the effects of bias in the filtered results. Formally, the global filter can be expressed as follows for Rician noise:

11 $${\hat{\mathbf{x}}} = {{\mathbf{W}}}{\mathbf{y}} = VST^{ - 1} (({\mathbf{V}}_{m} {\mathbf{E}}_{m}^{k} {\mathbf{V}}_{m}^{T} VST({\mathbf{y}},\sigma_{R} )),\sigma_{R} ) ,$$

where *VST*^−1^ denotes the inverse of *VST* and $\sigma_{R}$ is the standard deviation of the Rician noise. Thus, the noisy Rician data y is first stabilized by the VST and then filtered using the proposed global filter. The denoised results are finally obtained by applying an inverse VST to the denoising output.

It should be noted: firstly, since the global framework implemented the whole dataset to obtain the optimal similarity weight matrix, this scheme will be improvable for currently used patch-based denoising methods. Secondly, the computational burden of the proposed global framework is prohibitively high due to 3D structure of MRI dataset. As a result, we have implemented the Nyström method [19] in the following section in order to ameliorate this problem. We then introduce a k-means clustering which further improves the global framework to achieve applicable implementation for 3D MRI.

### K-means Nyström approximation for global filtering

As mentioned above, the proposed global filter demands extremely high computational and storage costs. Fortunately, as mentioned previously, the proposed global framework only requires a portion of the ‘best’ eigenvectors and eigenvalues for efficient matrix approximation, rather than computing every element of **W**. This strategy can be effectively achieved by the Nyström method.

The Nyström method offers an efficient way to generate a low-rank approximation of the original matrix from a subset of its columns [19, 25, 26]. Given a matrix **M**, the eigenvectors can be estimated numerically:

12 $${\mathbf{M}} = {\varvec{\upgamma \upvarepsilon \upgamma }}^{T} ,$$

where ${\varvec{\upgamma}} = [\gamma_{1} , \ldots ,\gamma_{n} ]$ and ${\varvec{\upvarepsilon}} = [\varepsilon_{1} , \ldots ,\varepsilon_{n} ]$ represent the eigenvector and eigenvalue of **M**, respectively. Nyström suggests [19] that instead of computing all of the entries in **M**, the ‘best’ eigenvectors can be estimated through a sampled subset of the whole dataset, in order to obtain an approximation for ${\tilde{\mathbf{M}}}$.

Let the *l* × *l* matrix **M**_1_ denote the similarity weights of voxels in the subimage **W**_1_, which samples *l* voxels from the entire image. The (*n* *−* *l*) × (*n* *−* *l*) matrix **M**_2_ denotes the similarity weights of voxels in subimage **W**_2_, which contains (*n* *−* *l*) unsampled voxels. The *l* × (*n* *−* *l*) matrix **M**_12_ denotes kernel weights between voxels in **M**_1_ and **M**_2_. The similarity matrix **M** can then be defined as.

13 $${\mathbf{M}} = \left[ \begin{aligned} {\mathbf{M}}_{1} \quad {\kern 1pt} {\kern 1pt} {\kern 1pt} {\mathbf{M}}_{12} \hfill \\ {\mathbf{M}}_{12}^{T} {\kern 1pt} {\kern 1pt} {\kern 1pt} \quad {\mathbf{M}}_{2} \hfill \\ \end{aligned} \right],$$

According to Nyström, the approximation of the ‘best’ eigenvectors of **M** can be defined as:

14 $${\tilde{\boldsymbol{\gamma}}} = \left[ \begin{array}{c}{\varvec{\upgamma}}_{1}\\ {\mathbf{M}}_{12}^{T} {\varvec{\upgamma}}_{1} {\varvec{\upvarepsilon}}_{1}^{ - 1} \end{array} \right],$$

where ${\mathbf{M}}_{1} = {\varvec{\upgamma}}_{1} {\varvec{\upvarepsilon}}_{1} {\varvec{\upgamma}}_{1}^{T}$ and the approximation of ${\tilde{\mathbf{M}}}$ can then be obtained as:

15 $${\tilde{\mathbf{M}}} = {\boldsymbol{\tilde{\gamma }\varepsilon }}_{1} {\tilde{\boldsymbol{\gamma }}}^{T} = \left[ \begin{array}{ll} {\mathbf{M}}_{1} & \qquad {\mathbf{M}}_{12} \\ {\mathbf{M}}_{12}^{T} &{\mathbf{M}}_{12}^{T} {\mathbf{M}}_{1}^{ - 1} {\mathbf{M}}_{12} \\ \end{array} \right]$$

It is evident that the large matrix **M**_2_ in (12) has been simplified to ${\mathbf{M}}_{12}^{T} {\mathbf{M}}_{1}^{ - 1} {\mathbf{M}}_{12}$. In this circumstance, the subset **M** is crucial to the accuracy of the matrix approximation and computational efficiency. Several methods have been proposed to define a sampling strategy for subset **M.** The most intuitive sampling method for Nyström approximation is uniform sampling in which columns are selected using a fixed probability distribution [25]. It was implemented successfully in natural scene imagery. However, since MRI datasets contain a large background area and the informative voxels are distributed in a concentrated manor, uniform sampling cannot select informative voxels effectively. Besides uniform sampling, the dataset can be sampled non-uniformly, in which sampling is biased towards selection of the most informative columns of the matrix. For example, column-norm sampling has been implemented to analyze a singular value decomposition approximation algorithm [27], while diagonal sampling has been used to limit reconstruction error in the Nyström method [28, 29]. Unfortunately, these distributions cannot be efficiently computed in practice, except for special cases in which the dataset coincides with the sampling distribution.

In addition to fixed sampling methods, various adaptive sampling schemes have also been proposed to produce efficient low-rank approximations. The sparse greedy matrix approximation [30, 31] involves matching a pursuit algorithm to a new sample, randomly selected from a subset in successive rounds. The incomplete Cholesky decomposition [28] generates low-rank factorization through adaptive selection of columns based on potential pivots. The K-means clustering method stores centroids, which are used to generate informative columns [32].

For an MRI dataset, the informative voxels are concentrated in only a portion of the columns and the number of background voxels is large. Motivated by this observation and the fact that k-means clustering can find a local minimum in the quantization error [33], we propose using centroid voxels obtained by k-means clustering as adaptive sampled voxels in the Nyström approximation. The reasons for choosing k-means clustering for adaptive voxel sampling are threefold and can be summarized as follows: firstly, the k-means clustering ensures most of the selected voxels are located in the informative parts of the dataset and excludes redundant sampling of voxels from the background area. Secondly, the centroids obtained by k-means clustering are distributed throughout the whole dynamic range, which is more representative of the whole dataset and thus improves the estimation accuracy. Thirdly, the k-means clustering itself has been proven to be more suitable for handling large datasets with minimal quantization errors [32]. Let *k* be the desired number of clusters: the larger *k*, the more accurate the approximation, together with higher computational burden. To explore the effectiveness of the proposed k-means clustering method, a quantitative comparison including uniform sampling and adaptive sampling, namely the sparse greedy matrix approximation and incomplete Cholesky decomposition, is proposed. The comparison was implemented using in-house MRI datasets, which included 35 different datasets from various parts of the human body. The performance of different sampling schemes using the Nyström approximation was evaluated using the relative accuracy defined in Ref. [19]:

16 $$\textit{Relative Accuracy} = \frac{{\left\| {{\mathbf{K}} - {\mathbf{K}}_{(r)} } \right\|_{F} }}{{\left\| {{\mathbf{K}} - {\tilde{\mathbf{K}}}_{(r)} } \right\|_{F} }} \times 100,$$

where **K** and **K**_(*r*)_ are the actual kernel and its exact rank-*r* approximation, *F* denotes the Frobenius norm. Using the Nyström approximation, the approximated kernel ${\tilde{\mathbf{K}}}_{(r)}$ can be reconstructed using *r* leading eigenvectors. Note that the relative accuracy is lower-bounded by 0 and will approach 1 as a good approximation. In our experiment, *r* is fixed at 100, which captures more than 90 % of the spectral energy in the MRI dataset.

As demonstrated in Table 1, the k-means method achieved the best performance among all algorithms, generating the most accurate approximation in nearly every setting. When the sampling rate increased up to 20 %, the random sampling method even outperforms some adaptive methods; this fact was also observed by Zhang et al. [32]. This suggests there is a trade-off between time and space requirements.

**Table 1:** Nyström reconstruction accuracy for various sampling methods for MRI datasets for three *l*/*n* percentages

l/n %	Uniform	ICL	SMGA	K-means
5	62.3 (0.6)	67.7(0)	73.8 (1.3)	*74.9 (0.8)*
10	73.6 (1.1)	78.2(0)	81.7 (1.5)	*86.2 (1.2)*
20	84.8 (1.7)	83.4(0)	89.6 (1.9)	*91.3 (1.1)*

Numbers in parenthesis indicate the standard deviations for 10 different runs for *l*. Numbers in italics indicate the best performance on each dataset

After approximation of the similarity matrix **M**, the denoising filter **W** can be obtained as a row-normalized kernel **M** as demonstrated in Eq. 4:

17 $${\mathbf{W}} = {\mathbf{D}}^{ - 1} {\mathbf{M}},$$

where ${\mathbf{D}} = diag\left[ {\sum\nolimits_{j = 1}^{n} {K_{1j} } ,\sum\nolimits_{j = 1}^{n} {K_{2j} } , \ldots ,\sum\nolimits_{j = 1}^{n} {K_{nj} } } \right]$. Since we have already estimated the ‘best’ eigenvectors of **M**, the symmetric positive definite matrix **W** can be estimated using the Sinkhorn algorithm [21]:

18 $${\mathbf{W}} = \left[ \begin{aligned} {\mathbf{W}}_{1} {\kern 1pt} {\kern 1pt} {\kern 1pt} \quad {\mathbf{W}}_{12} \hfill \\ {\mathbf{W}}_{12}^{T} {\kern 1pt} {\kern 1pt} {\kern 1pt} \quad {\mathbf{W}}_{2} \hfill \\ \end{aligned} \right],$$

Finally, **W** is orthogonalized to achieve the final denoising filter ${\tilde{\mathbf{W}}}$. Define:

19 $${\mathbf{O}} = {\mathbf{W}}_{1} + {\mathbf{W}}_{1}^{ - 1/2} {\mathbf{W}}_{12} {\mathbf{W}}_{12}^{T} {\mathbf{W}}_{1}^{ - 1/2} ,$$

where ${\mathbf{W}}_{1}^{ - 1/2}$ is the symmetric positive definite square root of ${\mathbf{W}}_{1}^{{}}$ and **O** can be eigen-decomposed as ${\mathbf{O}} = {\mathbf{V}}_{O} {\mathbf{E}}_{O} {\mathbf{V}}_{O}^{T}$. The orthogonalized ${\tilde{\mathbf{W}}}$ can then be expressed as:

20 $${\tilde{\mathbf{W}}} = {\tilde{\mathbf{V}}}{\tilde{\mathbf{E}}}{\tilde{\mathbf{V}}}^{T} .$$

where $${\tilde{\mathbf{E}}} = {\mathbf{E}}_{O}$$ and $${\tilde{\mathbf{V}}} = \left[ \begin{array}{c} {\mathbf{W}}_{1} \hfill \\ {\mathbf{W}}_{12}^{T} \hfill \\ \end{array} \right]{\mathbf{W}}_{1}^{ - 1/2} {\mathbf{V}}_{O} {\mathbf{E}}_{O}^{ - 1/2}$$.

This global filter ${\tilde{\mathbf{W}}}$ is approximated using the leading eigenvectors and eigenvalues and can be implemented for denoising using a small sampling rate which nearly matches the performance of the exact filter.

## Experiments

The proposed framework was evaluated using state-of-the-art algorithms in both synthetic and in vivo MRI datasets. In our proposed global framework, both the non-local means [2] and BM3D [8] were implemented as baseline kernels in spatial and transform domain respectively. It should be noted that any patch-based denoising method could be used in our proposed global denoising framework. The k-means clustering scheme was implemented for the Nyström approximation and the sampling rate was set to 0.0005 %, which was defined and discussed in the following section. The non-local means filter and BM3D were implemented for comparison and the parameters were set as indicated in [2] and [8], respectively.

Two open-access datasets, namely BrainWeb (simulated images) [34] and the *Internet Brain Segmentation Repository* (IBSR—real images) [35] were selected for comparison. In addition, a real MRI dataset containing the spinal cord and a real diffusion-weighted MRI dataset were utilized to evaluate the adaptability and robustness of the proposed framework.

**Fig. 1:**
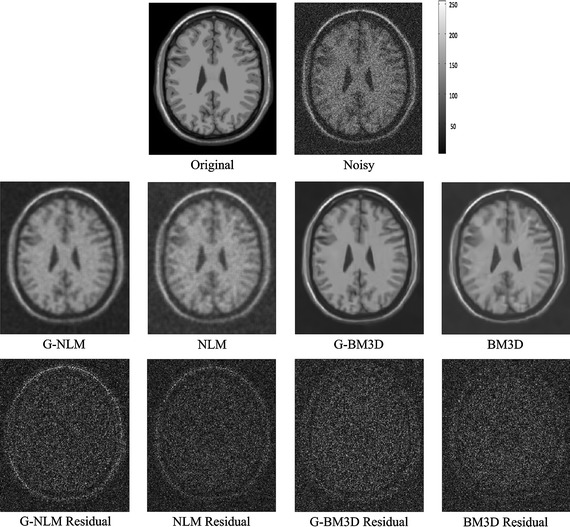
Denoising results for an axial slice of the T1w BrainWeb phantom (Rician noise level of 15 %). The* third row* demonstrates the absolute value of the residual for different methods

**Fig. 2:**
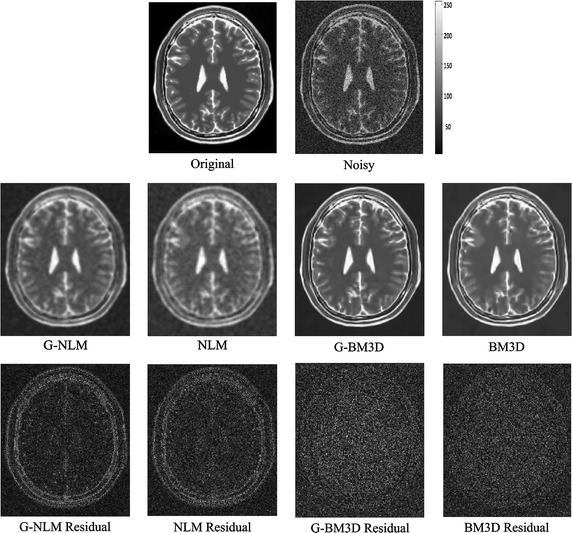
Denoising results for an axial slice of the T2w BrainWeb phantom (Rician noise level of 15 %). The* third row* demonstrates the absolute value of the residual for different methods

**Fig. 3:**
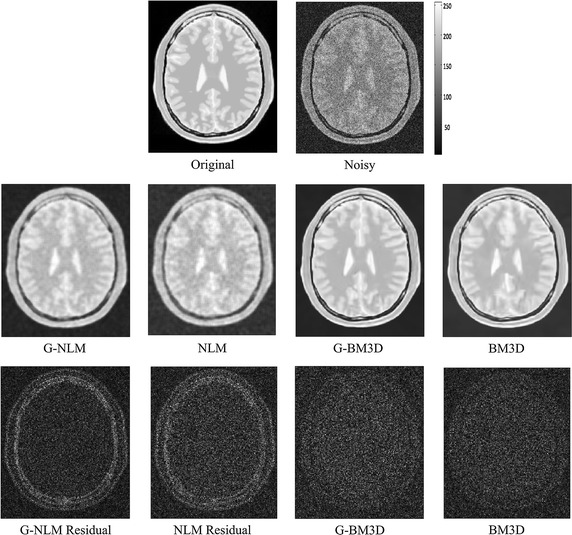
Denoising results for an axial slice of the PDw BrainWeb phantom (Rician noise level of 15 %). The* third row* demonstrates the absolute value of the residual for different methods

For the BrainWeb dataset, T1, T2, and PD weighted 3D MRI phantoms were used. Each of these phantoms had a voxel size of 1 × 1 × 1 mm and was 181 × 217 × 181 voxels in size. To evaluate the performance of the proposed framework, the same three phantom images corrupted by different levels of Rician noise (1–15 % of maximum intensity) were used and each image was denoised using the four denoising algorithms outlined previously. The visual quality, together with the residuals (rescale to 64 gray level for better discernment) of the T1, T2, and PD weighted datasets, are shown in Figs. 1, 2 and 3, respectively. The peak signal-to-noise ratio and structural similarity index [36] were selected for quantitative comparison (see Table 2).

**Table 2:** PSNR and SSIM of the compared methods for different MRI modalities and Rician noise levels

Noise level (%)			1	3	5	7	9	11	13	15
T1w	PSNR	NLM	36.74	31.07	29.27	28.03	26.86	25.72	24.63	23.46
		G-NLM	36.23	32.11	29.16	28.93	27.34	27.01	25.85	24.37
		BM3D	*43.40*	36.65	*33.48*	31.26	29.26	27.47	25.97	24.40
		G-BM3D	42.56	*36.78*	33.32	*32.23*	*30.67*	*29.26*	*26.78*	*26.12*
	SSIM	NLM	0.9700	0.9042	0.8666	0.8319	0.7968	0.7617	0.7275	0.6907
		G-NLM	0.9625	0.9041	0.8745	0.8437	0.8052	0.7861	0.7432	0.7231
		BM3D	*0.9904*	0.9632	0.9320	0.8993	0.8661	0.8326	0.8029	0.7697
		G-BM3D	0.9903	*0.9638*	*0.9362*	*0.9016*	*0.8795*	*0.8563*	*0.8247*	*0.7968*
T2w	PSNR	NLM	33.37	27.52	24.43	22.99	22.16	21.55	20.99	20.42
		G-NLM	33.43	27.51	25.12	23.37	22.06	21.87	21,34	20.64
		BM3D	42.54	*34.97*	31.68	29.50	27.77	26.34	24.87	23.50
		G-BM3D	*42.89*	33.67	*32.54*	*30.68*	*28.52*	*26.43*	*25.26*	*24.37*
	SSIM	NLM	0.9677	0.9051	0.8637	0.8259	0.7850	0.7467	0.7078	0.6731
		G-NLM	0.9658	0.9037	0.8764	0.8347	0.7938	0.7637	0.7286	0.7031
		BM3D	*0.9898*	0.9627	0.9334	0.9084	0.8768	0.8497	0.8201	0.7918
		G-BM3D	0.9884	*0.9638*	*0.9467*	*0.9128*	*0.8856*	*0.8643*	*0.8432*	*0.8169*
PDw	PSNR	NLM	34.81	30.11	27.55	25.85	24.74	23.95	23.35	22.75
		G-NLM	36.78	32.22	29.53	26.84	24.97	24.65	24.36	23.77
		BM3D	43.43	36.40	33.28	31.27	29.62	28.25	27.10	25.86
		G-BM3D	*43.56*	*37.37*	*34.56*	*32.78*	*30.62*	*30.17*	*28.96*	*26.77*
	SSIM	NLM	0.9728	0.9075	0.8401	0.7856	0.7435	0.7088	0.6742	0.6463
		G-NLM	0.9823	0.9234	0.8564	0.7958	0.7736	0.7297	0.7056	0.6573
		BM3D	0.9915	0.9665	0.9403	0.9144	0.8885	0.8678	0.8406	0.8163
		G-BM3D	*0.9927*	*0.9711*	*0.9568*	*0.9347*	*0.8982*	*0.8768*	*0.8543*	*0.8256*

Numbers in italics indicate the best performance on each dataset

**Fig. 4:**
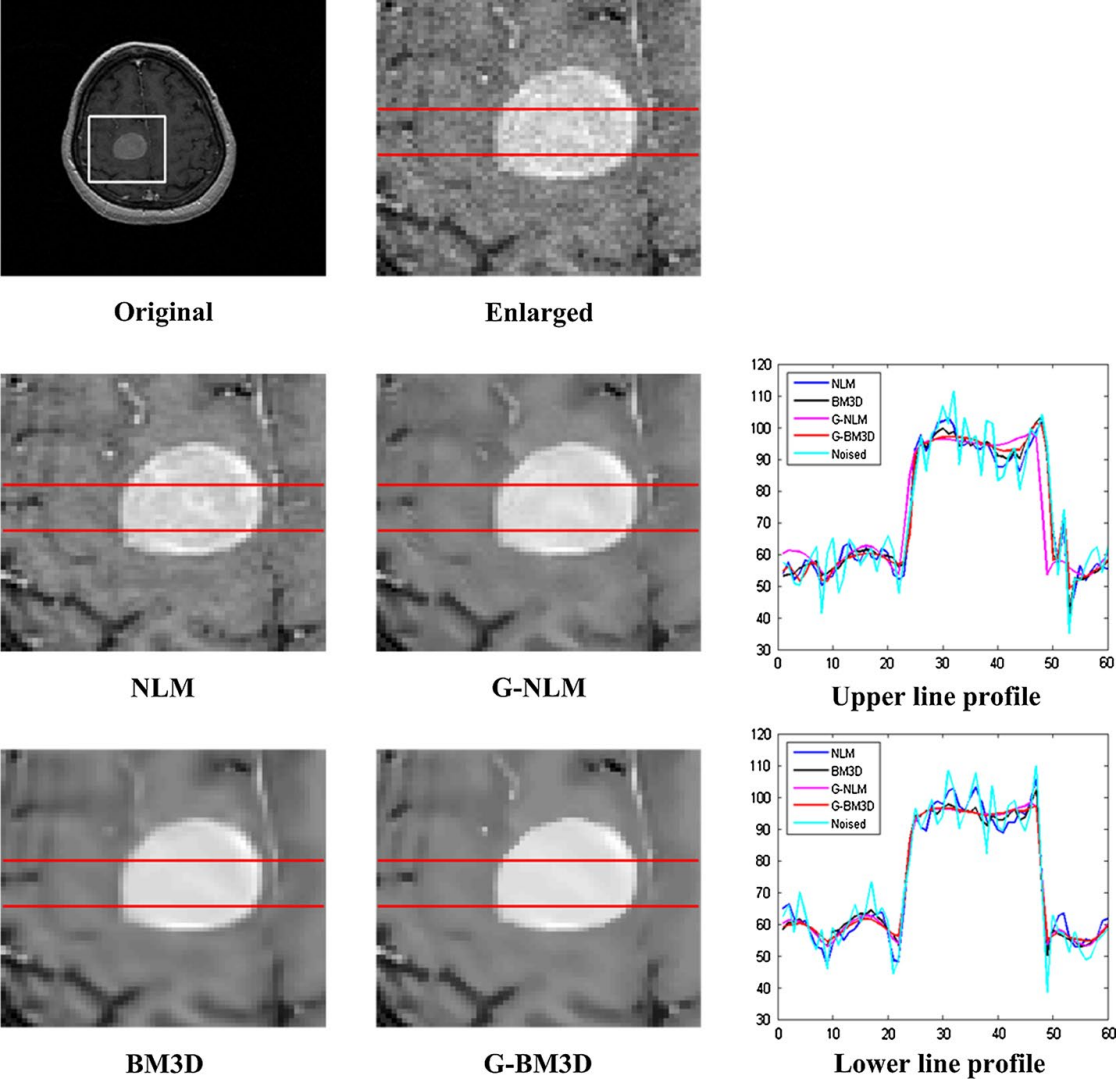
Denoising results for the IBSR dataset. From *top* to *bottom*: original volume and enlarged target tissue, denoising results of the enlarged target tissue using NLM, G-NLM and the profile of the* upper line*, denoising results for the enlarged target tissue using BM3D, G-BM3D and the profile of the *lower line*

For the IBSR dataset, 2 % Rician noise was added to achieve better discernment of the algorithm performance; the results are shown in Fig. 4. In addition to a visual comparison, the two horizontal profiles are also denoted.

**Fig. 5:**
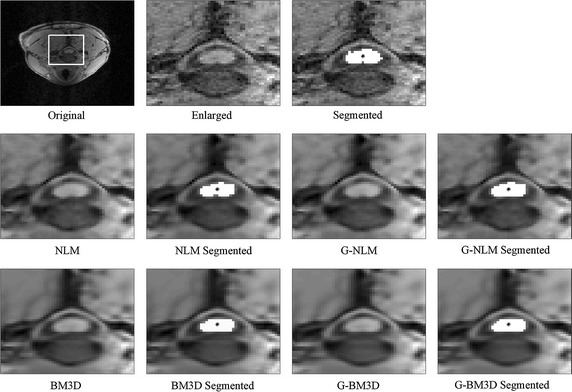
Denoising results for the real MRI dataset of the spinal cord. From *top* to *bottom*: original volume, denoised results and corresponding segmented results using NLM and the proposed global framework, denoised results and corresponding segmented results using BM3D and the proposed global framework

The real 3D MRI dataset for the spinal cord was acquired using a Siemens 3T scanner [TE = 4.7 ms, TR = 2040 ms, TI = 900 ms, voxel size = 1.0 × 1.0 × 1.0 mm, image size = 180 × 192 × 192, flip angle = 8°]. The Rician noise level was estimated to be around 3 % of the maximum gray level intensity using the method proposed by Aja-Fernández et al. [37]. Since the original dataset was already noisy, only the restored images are shown in Fig. 5. In addition to a visual comparison, the results of segmentation with a region-growing algorithm [38] are also provided. The seed point was manually selected in the center of the spinal cord area and is indicated with an asterisk.

**Fig. 6:**
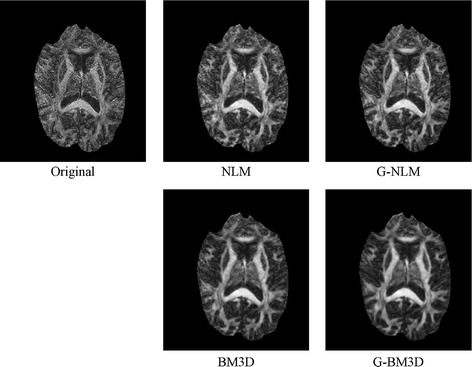
Denoising results for the FA map of the DWI dataset

**Fig. 7:**
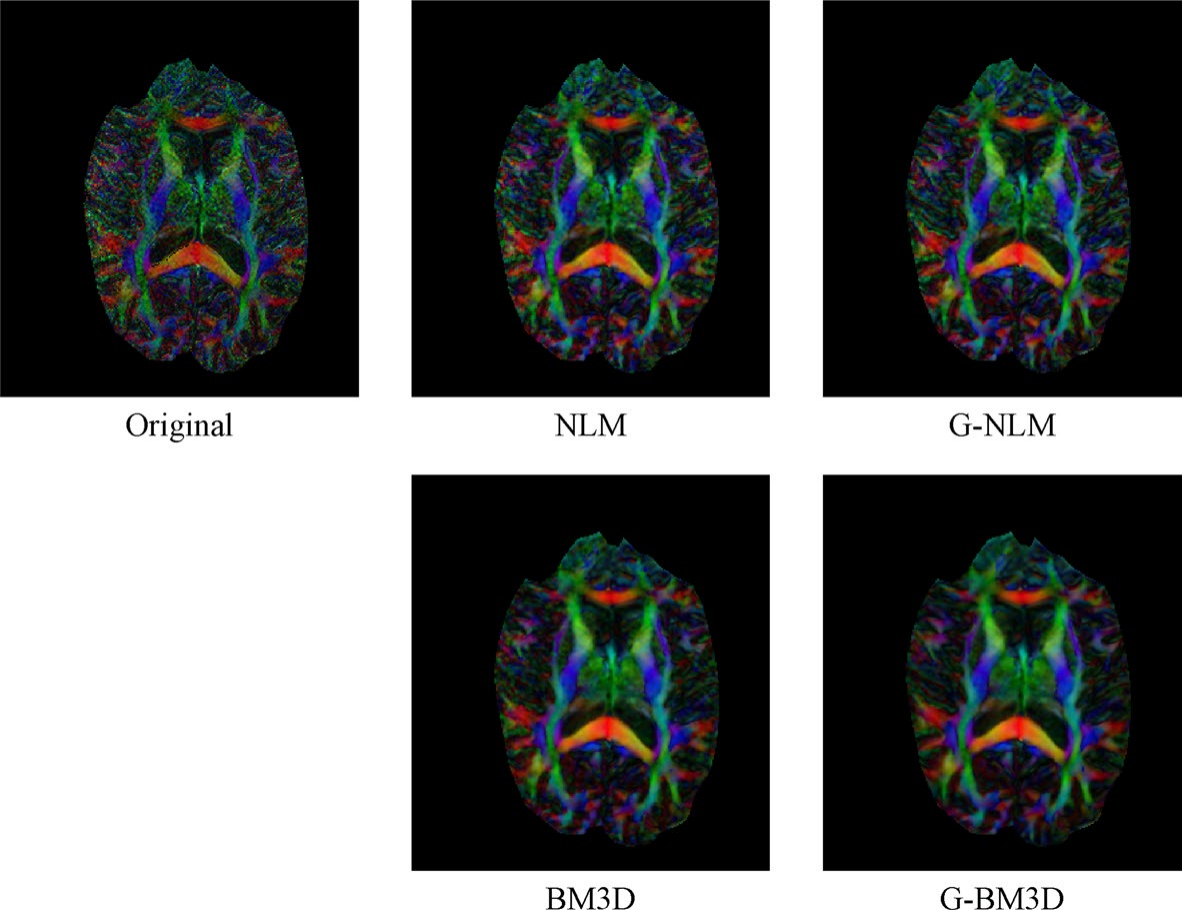
Denoising results for the principle direction weighted with FA of the DWI dataset

The real 3D diffusion-weighted imaging dataset was obtained using a 3T Philips Intera Achieva MRI scanner (Best, The Netherlands), using an eight-element SENSE coil and a single shot echo-planar pulsed gradient spin-echo imaging sequence. Diffusion weighting was performed along 32 non-collinear directions with a value of 1000 s/mm^2^ (matrix size = 128 × 128, field of view = 256 × 256 mm^2^, TE = 60 ms, TR = 10 s, thickness = 2 mm, gap = 0, SENSE factor = 2). Diffusion tensor imaging was estimated from diffusion-weighted imaging data using a linear least-squares fitting procedure [20]. From this, the fractional anisotropy and principle direction (PD) weighted with fractional anisotropy were computed; the results are shown in Figs. 6 and 7, respectively.

## Results

Figures 1, 2 and 3 demonstrate visual evaluation of the denoising results using different MRI modalities with a 15 % noise level. It can been observed that the proposed global denoising framework improves patch-based methods (whether non-local means or BM3D) in different MRI modalities. Specifically, denoising results for the global filter feature less intensity oscillation in homogenous areas compared with the original patch-based method. This finding can be more clearly observed in residual images. Moreover, the global filter can improve denoising of patch-based methods when there are high levels of noise. This finding is also in agreement with the quantitative comparison shown in Table 1, which shows that the peak signal-to-noise ratio and the structural similarity index achieved by the global framework always achieve the best results. Finally, both edge and feature preservation can also benefit from this global filter. It can clearly be observed that the residual images in the global filter contain less structural information compared with residuals from the original NLM or BM3D. This coincided with all modalities including T1w, T2w, and PDw.

Figure 4 shows denoising results using the proposed global filter with a real MRI dataset from IBSR. Since the original images already contain noise, neither ideal residual images nor quantitative results can be obtained. As demonstrated with the synthetic dataset, the proposed global filter attains good results in terms of both noise removal and edge preservation. Similar to the results with the synthetic dataset, the denoising results from the global filter demonstrate a stable intensity in the central region of the target tissue and sharper edges. This can be better observed from line profiles which have smaller amplitude differences near the edges and attenuate the visible artifacts manifested in the image.

Figure 5 demonstrates the consistency of the proposed global framework for a real spinal cord MRI dataset. Upon close inspection of the central area, it can be seen that the denoised results using the proposed method include a more explicit boundary, which matches the results obtained with the synthetic datasets. The segmentation results in the denoised image, which produce smoother contours in the spinal cord image, may be beneficial to further applications.

Figures 6 and 7 demonstrate the effect of shape and orientation information on the diffusion tensor computed using the denoised results in diffusion-weighted imaging. The original image of the PD orientation map and the fractional anisotropy contain obvious artifacts. Compared with the results obtained using the non-local means and BM3D methods, the proposed global framework exhibits visually significant improvements in both PD and fractional anisotropy. For the two methods using the proposed global framework, the results appear smoother and preserve more fine structural detail.

## Discussion

We proposed a global filtering framework for 3D MRI. Compared with commonly used patch-based methods, the proposed global filter uses all of the voxels in an input image to denoise every single voxel. Since the involvement of all voxels in the 3D MRI dataset results in a prohibitively high computational burden, we implemented a k-means clustering Nyström method to produce a low-rank approximation in order to achieve a viable algorithm.

The primary concern for efficiently generating a low-rank matrix approximation using the Nyström method is the sampling scheme. In this study, a k-means clustering algorithm was proposed as an adaptive sampling scheme, which effectively improved both estimation accuracy and computational efficiency. Compared with commonly-used fixed sampling schemes, which acquire voxels from the whole dataset, the k-means clustering method excludes redundant sampling of background voxels which form a large portion of the MRI dataset and concentrates the sampling in the informative area. Moreover, since the k-means clustering obtained centroids covering the whole dynamic range of gray levels, it ensures a fully representative selection of voxels for the Nyström approximation. From this point, a more elegant clustering algorithm, together with more effective clustering criteria, retains the potential for further improvements.

**Fig. 8:**
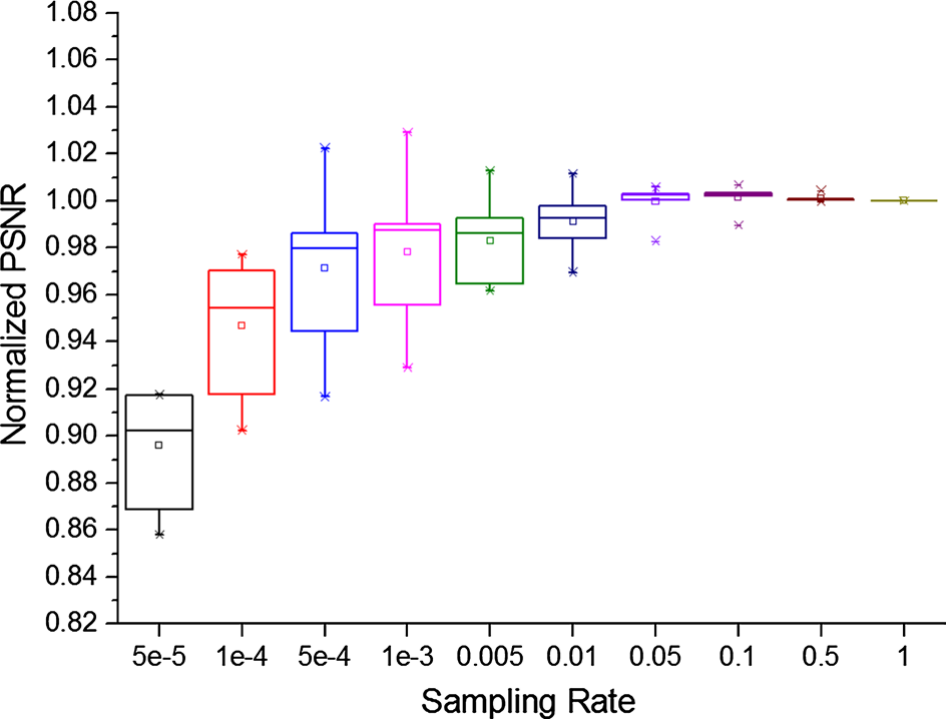
Comparison of the denoising performance of the exact and approximated filter for BrainWeb T1w, T2w, PDw phantom, and real MRI and DWI datasets. Note that the dataset is corrupted with 20 % Rician noise, the sampling rate is computed as p = n/100, and PSNR is normalized by the results of the exact filter

Sampling rate is another crucial factor which compromises both the approximation accuracy and computational complexity. As demonstrated in Table 1, a higher sampling rate ensures a more accurate approximation. In addition, the proposed k-means clustering sampling scheme achieved a more accurate approximation result, as a result of more effective sampling of the informative voxels. The denoising performance between the exact and approximated filter is compared in Fig. 8. The T1, T2, and PD weighted MRI phantom from the BrainWeb dataset, together with in vivo MRI and diffusion-weighted imaging datasets, were involved in this comparison. The peak signal-to-noise ratio for each dataset was normalized by the results of the exact filter. It can be seen that the discrimination of denoising performance at different sampling rates is more moderate than in the matrix approximation. Although there are slight improvements as the sampling rate is increased, a corresponding computational burden led to the selection of 0.0005 as a reasonable sampling rate. The results also suggest that the proposed Nyström method with k-means clustering can achieve nearly the same performance as the exact filter, using an applicable sampling rate for 3D MRI. This is likely because information oriented k-means clustering can provide plenty of landmark voxels for sampling, thereby improving the sampling efficiency.

Finally, the runtime for the proposed global filter, as demonstrated in Table 3, is reasonable for a typical 181 × 217 × 181 3D MRI dataset using an Intel Core i7 CPU platform and matlab R2013b. With the above setting, the proposed G-NLM and G-BM3D required 18 and 8 min, whereas NLM and BM3D required 40 and 14 min, respectively. However, parallelizing the computation could be implemented to further reduce processing time.

**Table 3:** Running time (in seconds) of the compared methods

Volume size	NLM	G-NLM	BM3D	G-BM3D
181 × 217 × 181	2412.5	1091.3	845.9	487.6

## Conclusions

We have proposed a global filtering framework for a 3D MRI dataset, which used the whole dataset to denoise each voxel. Due to its size, the large global denoising weight matrix was low-rank approximated using the Nyström method. In addition, k-means clustering was implemented as an adaptive sampling scheme to further improve the approximation accuracy and computational efficiency. The proposed global filtering framework is not restricted to any specific patch-based algorithm and our results show that it can be used to improve most patch-based methods.
